# Steroid Use for Established Bronchopulmonary Dysplasia: A Systematic Review and Meta-Analysis

**DOI:** 10.3390/children12091238

**Published:** 2025-09-16

**Authors:** Maria Pierro, Roberto Chioma, Krzysztof Włodarczyk, Margit Benke, Kaushik Mangroo, Maria Chiara Vetrano, Kinga Zielińska, David O’Keeffe, Joanna Seliga-Siwecka, Helen Purtill, Niazy Al-Assaf, Eduardo Villamor, Roy K. Philip

**Affiliations:** 1 Division of Neonatology, Department of Paediatrics, University Maternity Hospital Limerick, V94 C566 Limerick, Ireland; margitbenke@gmail.com (M.B.); kaushik.mangroo@monashhealth.org (K.M.); david.okeeffe@nmh.ie (D.O.); niazy.al-assaf@hse.ie (N.A.-A.); roy.philip@hse.ie (R.K.P.); 2 Neonatology Unit, Ospedale Isola Tiberina, Gemelli Isola, 00186 Rome, Italy; roberto.chioma.fw@fbf-isola.it; 3 Main Library, Medical University of Warsaw, 02-091 Warsaw, Poland; krzysztof.wlodarczyk@wum.edu.pl; 4 Department of Pediatrics-Neonatology, Ferrara University, 44121 Ferrara, Italy; vetranomariachiara@gmail.com; 5 Neonatal and Intensive Care Department, Medical University of Warsaw, 02-091 Warsaw, Poland; kinga.zielinska@szpitalkarowa.pl (K.Z.); seliga.joanna@gmail.com (J.S.-S.); 6 Department of Mathematics and Statistics, University of Limerick, V94 T9PX Limerick, Ireland; helen.purtill@ul.ie; 7 Department of Pediatrics, University of Limerick School of Medicine, V94 T9PX Limerick, Ireland; 8 Division of Neonatology, MosaKids Children’s Hospital, Maastricht University Medical Center (MUMC+), School for Oncology and Developmental Biology (GROW), 6229 Maastricht, The Netherlands; e.villamor@mumc.nl

**Keywords:** bronchopulmonary dysplasia, postnatal steroids, meta-analysis

## Abstract

**Highlights:**

Steroids are largely used for established bronchopulmonary dysplasia (BPD) to wean off oxygen or respiratory support. However, data from the literature are scarce and, to our knowledge, have never been systematically collected and analyzed. This systematic review and meta-analysis gathers all the available evidence on steroids for established BPD.

**What are the main findings?**

- Steroids have a good rate of success in terms of oxygen decrease. However, more clinically relevant outcomes, such as the total duration of supplemental oxygen, length of hospital stay, and mortality, are not affected by the use of steroids after 28 days of life. No significant side effects are reported in the literature. No clinical variables analyzed in the available studies were associated with the success of the therapy.
- Despite steroid treatment being regularly used for established BPD, only one study reported the incidence of this treatment exclusively after 28 days of life. Two independent, high-level neonatal centers describe steroid administration as standard treatment for severe BPD in order to avoid missing potential responders.

**What is the implication of the main finding?**

- Identifying patients who would benefit from steroid treatments is a priority in order to design statistically and clinically relevant studies.
- The observed drug safety profile of steroids supports the design of future trials in patients with established BPD.

**Abstract:**

**Introduction:** Evidence on steroid treatment for established bronchopulmonary dysplasia (BPD) is sparse. To our knowledge, a systematic review has never been conducted on this topic. This meta-analysis aims to synthesize available evidence for the use of postnatal steroids to treat established BPD. **Methods:** MEDLINE, Embase, Cochrane databases, and gray literature sources were searched without time or language restrictions until October 2024. We included randomized and non-randomized trials (analyzed separately) that evaluated postnatal steroids started from 28 days of life in preterm infants diagnosed with BPD. Certainty of evidence was assessed using the GRADE approach. **Results:** The search retrieved 9113 records, and 20 studies were included. Meta-analysis of the RCTs demonstrated that steroids significantly reduced oxygen requirement (daily mean difference of 1.6%, 95% CI 0.25–2.95), but the analysis did not identify significant differences in total duration of supplemental oxygen, length of stay, or mortality (moderate quality). From a safety perspective, steroids resulted in a transient increase in systemic blood pressure (mean difference of 6.8 mmHg, 95% CI 4.6–8.9) (moderate quality). Weight gain during treatment was lower in the systemic steroid group (−9.2 g/day, 95% CI −11.7 to −6.8) (moderate quality), although overall growth was reported as equal (2.4 g/day, 95% CI −0.3 to 6.3) (moderate quality). One retrospective study reported the incidence of steroid treatment among infants with established BPD (any definition) to be as high as 36%. Two single-arm studies reported a prolonged high-dose systemic steroid regimen as the routine treatment strategy for severe established BPD. **Conclusions:** Moderate quality of evidence suggests that steroid treatment cannot be recommended as standard of care for established BPD. However, corticosteroids are often used to this end. Large-scale RCTs designed to treat BPD are urgently needed. Furthermore, careful consideration for patient selection and compliance with GRADE methodology is essential.

## 1. Introduction

Bronchopulmonary dysplasia (BPD) is a chronic respiratory condition that affects 30 to 60% of preterm infants born before 32 weeks and 50 to 90% of infants born before 28 weeks of gestation, depending on the adopted definition [1,2,3]. BPD has a multifactorial pathogenesis resulting from the pathological reparative response of the developing lung to both prenatal and postnatal injury [4,5].

The clinical definitions and categorizations of BPD have evolved over time, and they are primarily based on the need for supplemental oxygen or respiratory support at 28 days of life and/or 36 weeks’ post-menstrual age (PMA) [6,7,8]. These definitions, based on components of care, do not fully capture the heterogeneity of BPD, a condition with multiple phenotypes reflecting different pathophysiological mechanisms, clinical presentations, and treatment responses [9].

Postnatal steroid treatment within the first two weeks of life has shown the best efficacy in preventing BPD [10]. However, early steroid use has been limited due to concerns of possible long-term neurodevelopmental effects [11,12]. Conversely, there is little information on the use of steroids to treat BPD once the condition is already established. To the best of our knowledge, this topic has not been systematically examined.

Here, we report the results of a systematic review and meta-analysis on the effects of steroid treatment in preterm infants suffering from established BPD.

## 2. Methods

### 2.1. Inclusion Criteria

This systematic review included randomized trials, case–control studies, cohort studies, and case series that evaluated the use of postnatal steroids in preterm infants <32 weeks of gestation and diagnosed with established BPD [6]. Studies were included if steroid treatment was started 28 days of postnatal age, with no restrictions regarding formulation, dosage, or route. All definitions of BPD were included. Placebo or no treatment was considered the comparator. Also, studies with no control group (single-arm studies) were included in the systematic review but not in the meta-analysis.

Studies comparing different steroid strategies after 28 days of life or comparing treatment after 28 days of life to earlier treatment were also included and analyzed separately.

### 2.2. Primary and Secondary Outcomes

The primary outcome was mortality within 1 year of life. A full description, definition, and references of the secondary outcomes can be found in the published protocol of this systematic review [13]. Briefly, we divided outcomes into efficacy and safety outcomes.

#### 2.2.1. Efficacy Outcomes

1. Short-term respiratory outcomes: Respiratory support step-down and oxygen decrease during treatment.
2. Medium-term outcomes: Total duration of oxygen dependency, length of stay, requirement for supplemental oxygen on discharge or home ventilation through a tracheostomy, and feeding difficulties.
3. Long-term outcomes: Duration of home oxygen use, number and severity of respiratory symptoms, hospital readmission for any cause and respiratory causes, and function and exercise tests.

#### 2.2.2. Safety Outcomes

Somatic growth, development of systemic hypertension, incidence of infections, blood sugar or electrolyte imbalance, incidence of secondary adrenal insufficiency, incidence and severity of osteopenia of prematurity, gastrointestinal bleeding and/or gastrointestinal perforation, neurodevelopmental delay.

### 2.3. Search Strategy

MEDLINE, Embase, Cochrane databases, and gray literature sources were searched without time or language restrictions from January 1966 to April 2022. A further update of the search was performed up to October 2024. The search strategy is fully reported in the Supplementary Materials.

### 2.4. Data Management

Literature search results were uploaded to Distiller Systematic Review (DistillerSR^®^) software (Ottawa, ON, Canada), an Internet-based software program that facilitates the study selection process (https://www.distillersr.com/products/distillersr-systematic-review-software, last access date 23 March 2023).

### 2.5. Study Selection Process

The articles were split into two sequential groups for feasibility reasons. Each group of articles was screened by two reviewers; thus, four reviewers were involved in the article selection process. Two independent reviewers screened article titles and abstracts in parallel using an initial screening questionnaire (first-level screening). Subsequently, full-text screening for all the articles retained after the first-level screening was conducted against our eligibility criteria (second-level screening) and reasons for exclusion were recorded. Any disagreement was settled by consensus, and when not possible, a third author was contacted for resolution.

Where mixed data were reported in the studies, only the patients who strictly met the inclusion criteria and were reported separately were included in the meta-analysis. If only mixed data were reported and the authors could not provide separate data, the study was excluded. No additional data that could be included in the meta-analysis were made available by contacting the authors.

### 2.6. Data Extraction

Data extraction forms were developed a priori and pilot-tested using a standardized extraction form on DistillerSR^®^. Two independent reviewers performed data extraction using a single charting and audit approach with the quality control function in DistillerSR^®^. Each reviewer charted half of the articles and audited the other half of their group of articles. In cases of reviewer disagreement, a third reviewer was consulted.

Results were discussed, and the data extraction form was updated iteratively to include aspects of treatment that were not listed a priori.

### 2.7. Data Analysis

Randomized and non-randomized studies were analyzed separately. Non-randomized studies were analyzed for outcomes that could not be meta-analyzed in randomized control trials (RCTs).

For dichotomous outcomes, the odds ratio (OR) for non-randomized studies and the risk ratio (RR) for randomized studies with 95% confidence intervals (CIs) were calculated from the data provided in the studies. For continuous outcomes, the standardized mean differences (MDs) and 95% CIs were calculated.

When studies reported continuous variables as medians and ranges or interquartile ranges, we estimated the means and standard deviations by using the method by Wan et al. [14].

All analyses were performed with Comprehensive Meta-analysis software V2 (Biostat, Inc., Englewood, CO, USA). The analyses were conducted with the random-effect model.

Statistical heterogeneity was assessed by Cochran’s Q statistic and by the I^2^ statistic.

Sensitivity analysis was performed to examine the relative contribution to heterogeneity of each study in a meta-analysis by removing one study at a time [15].

### 2.8. Subgroup Analysis

We planned to perform subgroup analysis based on a priori determined covariates of interest (severity of the disease based on oxygen support or BPD definition, type of steroid used, route of administration, duration and frequency of treatment, other ongoing treatments, previous treatment with steroids before 28 days of life, and definitions of established BPD). However, given the paucity of studies, only subgroup analysis based on the route of steroids (inhaled versus systemic) was feasible.

### 2.9. Risk of Bias Assessment

Methodological quality of the studies included in the meta-analysis was assessed by two authors independently using the ROBINS-I (Risk Of Bias In Non-randomized Studies—of Interventions) Scale for non-randomized studies and the Cochrane risk-of-bias tool for randomized trials. Discrepancies during assessment of the risk of bias were resolved by author discussion, and a third independent author was involved as required to reach consensus.

We defined the risk of bias for each individual outcome included in the meta-analysis.

### 2.10. GRADE Assessment and Display of the Results

The quality of evidence was rated according to the GRADE methodology [16]. Publication bias was not investigated because a low number of studies entered the meta-analysis.

All outcomes that reached a significance level above 3 (important and critical to decision making) were analyzed and tabulated in the summary of findings tables. The importance of the outcomes was rated by two authors independently. Inconsistencies were solved by discussion. Summary of findings tables and GRADE evidence profile tables with specification for subgroup analysis are reported in the Supplementary Material (Tables S1 and S2).

### 2.11. Protocol Registration, Amendments, and PRISMA Statement

**Figure 1:**
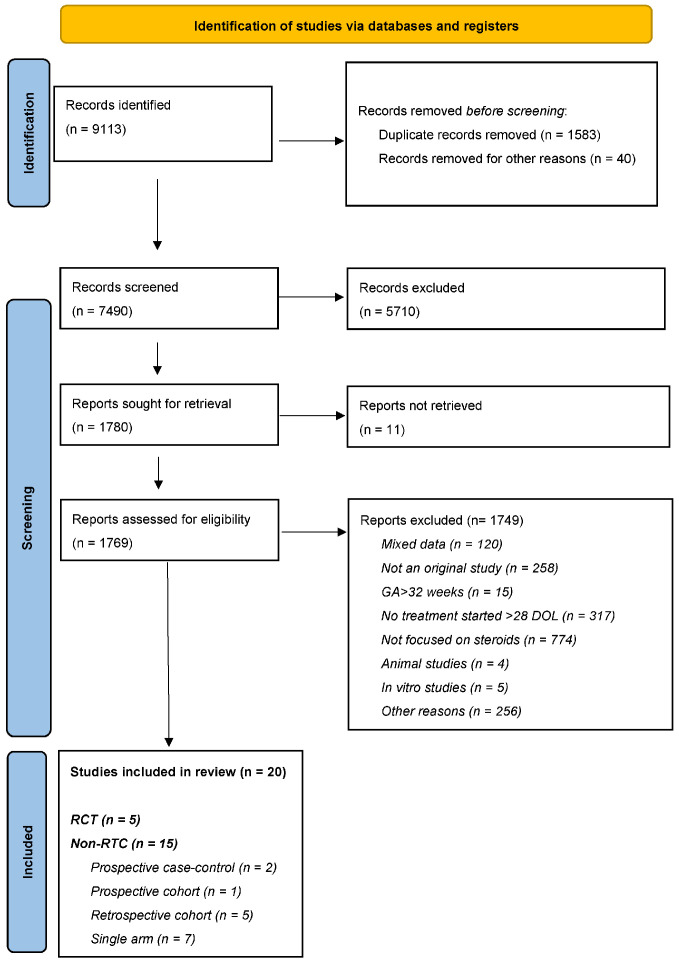
PRISMA Flowchart. Abbreviations: day of life (DOL), gestational age (GA), and randomized controlled trials (RCTs).

The protocol is registered on the PROSPERO international prospective register of systematic reviews (registration number CRD42021218881) and was previously published [13]. Amendments to the protocol are reported in the Supplementary Materials. The protocol design followed the PRISMA-P guideline. The meta-analysis was created and reported according to the PRISMA guidelines. The PRISMA flowchart is reported in Figure 1.

## 3. Results

### 3.1. Study Description

The initial search retrieved 9113 records, and 7490 records were retained after duplication removal. Results of the first- and second-level screenings and the design of the included studies are reported in the PRISMA flowchart in Figure 1. Further to the screening process, 20 studies met the inclusion criteria for this systematic review [17,18,19,20,21,22,23,24,25,26,27,28,29,30,31,32,33,34,35,36]. Characteristics of the included studies are reported in Table 1 and Table 2.

Two RCTs treated infants exclusively after 36 weeks PMA and did not assess common outcomes [19,21]. Among the non-RCTs with control groups, two studies investigated the use of steroids started exclusively after 36 weeks PMA. One study focused on the use of inhaled steroids [29], and the other study focused on systemic steroids [22]. Two single-arm studies treated infants after 36 weeks of PMA.

During the screening process, 120 studies were excluded because they included and analyzed a mixed set of patients with regard to treatment timing, i.e., treated before and treated after 28 days of life.

Interestingly, only one study provided epidemiological information on the exposure of infants with established BPD to steroids [22]. The single-center retrospective analysis of a prospective database by Bhandari and colleagues [22] reported treatment with systemic steroids in 36% of 385 preterm infants with moderate-to-severe BPD (requiring oxygen after 36 weeks PMA) born between 2000 and 2004 [22].

One large study on a UK national database reported epidemiological data on the use of different steroid formulations in preterm infants [37]. This study could not be included in the analysis, as the criteria for treatment timing were not met for all the steroid formulations. While descriptive details were provided separately, all steroid formulations were analyzed together with regards to the effect on the outcomes of interest for this systematic review. No epidemiological data specific to infants treated after 28 days of life could be derived either.

### 3.2. Efficacy and Safety of Steroids for Established BPD: Evidence from RCTs

Outcomes are presented in chronological clinical order and synthetized based on the GRADE informative statements to communicate the findings of systematic reviews of interventions [38].

#### 3.2.1. Efficacy Outcomes

##### 3.2.1.1. Steroid Treatment After 28 Days of Life Probably Improves Short-Term Respiratory Outcomes

The meta-analysis of the RCTs on systemic steroids [17,18] demonstrated a statistically significant improvement in oxygen reduction during treatment. The quality of evidence was rated as moderate through the GRADE assessment for this outcome (Table 3).

##### 3.2.1.2. Steroid Treatment After 28 Days of Life Probably Leads to No or Little Effect on Medium-Term Efficacy Outcomes

In the meta-analysis of RCTs, duration of oxygen dependency, length of stay, and mortality before discharge were not affected by the use of either systemic or inhaled steroids. The quality of evidence was rated as moderate through the GRADE assessment for these outcomes (Table 3).

##### 3.2.1.3. The Evidence Is Not Enough to Conclude on Long-Term Efficacy Outcomes Following Steroid Commencement After 28 Days of Life

No RCTs reported on mortality after discharge. Two RCTs investigated the effects of inhaled steroids on long-term respiratory outcomes after discharge [19,21]. Unfortunately, each study reported on different outcome measures; therefore, they could not be meta-analyzed. One of the two studies treated infants from 36 weeks PMA for 3 months and reported a lower number of respiratory related re-hospitalizations, re-hospitalization days, and post-discharge additive steroids in the treated group compared with the placebo group [21].

The other RCT treated infants from 36 weeks PMA until 1 year of age and failed to report a significant difference between treatment and placebo groups in the incidence of any respiratory symptom [19].

No studies reported on exercise or function tests.

#### 3.2.2. Safety Outcomes

No RCTs reported on long-term neurological and neurodevelopmental outcomes.

##### 3.2.2.1. Steroid Treatment After 28 Days of Life Probably Increases Blood Pressure

The meta-analysis of the two RCTs that evaluated blood pressure [20,21] found that blood pressure was significantly lower in the control group compared with the infants treated with inhaled steroids (Table 3). However, both studies reported that blood pressure normalized by the time of discharge in the treatment group and that no medication for hypertension was needed during admission. The quality of evidence was rated as moderate through the GRADE assessment for this outcome (Table 3).

Meta-analysis could not be run for systemic steroid administration. Harkavy and colleagues reported that 67% of the infants in the treatment group vs. 25% of the infants in the placebo group developed hypertension that responded well to standard medications [17]. However, no blood pressure values were reported. No patients were hypertensive at discharge. Noble-Jameson and colleagues do not mention recording blood pressure during treatment [18].

##### 3.2.2.2. Systemic Steroid Treatment After 28 Days of Life Probably Decreases Weight Gain During Treatment

Meta-analysis of the RCTs on systemic steroids showed that weight gain during treatment was lower in the steroid group (−9.2 g/day, 95%CI −11.7 to −6.8) (moderate quality of evidence). The study by Noble–Jamieson found that the increase in occipital frontal circumference (OFC) was similar between groups, suggesting adequate brain growth.

##### 3.2.2.3. Steroid Treatment After 28 Days of Life Probably Has Little or No Effect on Overall Weight Gain During Treatment

Meta-analysis of the RCTs shows that overall growth is not affected by steroid treatment (moderate quality of evidence). Subgroups analysis confirmed the finding for inhaled steroids (moderate quality of evidence). Subgroup analysis for systemic steroids could not be performed, as only one study reports on overall growth, detecting similar growth for steroid and placebo groups [17].

##### 3.2.2.4. Quality of Evidence Is Too Low to Make Any Assumption on the Effects of Steroid Treatment After 28 Days of Life with Regards to Adrenal Axis Balance or Hyperglycemia

The meta-analysis found no association between inhaled steroid use and adrenal insufficiency (Table 3). The quality of the evidence was rated as very low due to very serious inconsistency and serious imprecision (Table 3). Both studies investigated adrenal insufficiency by testing the urine cortisol/creatinine ratio. It appears that this heterogeneity could be related to the extremely high standard deviation in the results of the cortisol/creatinine ratio reported by Kugelman and colleagues [21]. On the contrary, the study by Dugas and colleagues [20] reports a lower variability in the cortisol/creatinine ratio results, identifying a non-significant trend towards lower values in the treated group.

The meta-analysis for hyperglycemia found no association with steroid treatment. However, the quality of evidence was rated as very low due to serious inconsistency and very serious imprecision (Table 3). The high heterogeneity could not be explained. One of the studies on systemic steroids reported a higher rate of hyperglycemia in the steroid vs. placebo groups (89% vs. 8%) [17], while the other RCT reported no difference in the incidence of hyperglycemia between groups [18]. The RCT on inhaled steroids reported no difference in the incidence of hyperglycemia [20].

##### 3.2.2.5. Steroid Treatment After 28 Days of Life May Lead to Little or No Effect on Infection Risk

Administration of steroids was not associated with infections during treatment. The quality was rated as moderate due to serious imprecision (Table 3).

### 3.3. Efficacy and Safety of Steroids for Established BPD: Additional Information from Non-RCTs

One retrospective study reported no differences in mortality at 24 months with inhaled corticosteroids in chronically ventilated preterm infants [29]. The same study found no association between late inhaled steroid exposure and neurodevelopmental impairment at 24 months corrected age.

One retrospective cohort study found that total cerebral, cortical, and cerebellar tissue volume and subcortical gray matter size in the dexamethasone-treated group were, respectively, 10.2%, 8.7%, 20.6%, and 19.9% smaller compared with the untreated group (Table 4). However, the profile of infants in the two groups were significantly different; steroid-treated infants were smaller, sicker, and ventilated for a longer period of time compared with controls. Logistic regression addressed only a few of these factors. In particular, the duration of mechanical ventilation was not included in the analysis [28].

No additional outcomes could be meta-analyzed for non-RCTs.

### 3.4. Efficacy and Safety of Steroids for Established BPD: Single-Arm Studies

The short-term respiratory benefit was uniformly described in the included single-arm studies. In addition to the reduction in the fraction of inspired oxygen (FiO_2_) described by RCTs, five studies reported increased lung compliance and decreased airway resistance, respiratory rate, mean airway pressure, oxygenation index, and pulmonary severity score [30,31,33,35,36].

Two retrospective studies reported a prolonged four-week prednisolone course as standard treatment for patients with severe BPD transferred to their level IV NICUs in the USA [30,31]. One described an improvement in oxygen need, but not in respiratory support step-down [30]. In the other one, 32% of the patients experienced a decrease in respiratory support during the four weeks of treatment [31].

No study, independently from the design, reported on the development of electrolyte imbalance, incidence and severity of osteopenia of prematurity, gastrointestinal bleeding, and/or gastrointestinal perforation.

### 3.5. Factors Associated with the Success and Safety of Steroid Treatment After 28 Days of Life

The success of steroid therapy had a median of 70.8% (range of 33–100). However, the criteria for defining the success of the therapy varied among the studies (Table 4).

Three studies investigated possible factors involved in the success of systemic steroid treatment for established BPD [22,24,34]. Five factors, gestational age (GA), PMA, infant sex, days at treatment, and partial carbon dioxide pressure (pCO_2_), could enter the meta-analysis. The only significant factor associated with the success of the therapy was earlier treatment initiation (70 ± 19 vs. 75.5 ± 25 days; *p* = 0.019) (Table S3). There was a trend towards lower therapeutic success when pCO_2_ was higher, but this did not reach statistical significance (Table S3).

### 3.6. Repeat Course of Steroids

With regards to repeat courses of steroids after 28 days of life, one small retrospective single-arm study reported a 93% success rate of a repeat dose of dexamethasone to facilitate extubation [32]. The study also found a significantly higher ventilation/perfusion ratio after 72 h of treatment and a significant decrease in FiO_2_ within nine days after starting the second course [32]. On the contrary, a cohort study reporting on the overall effectiveness of repeat dexamethasone treatment to facilitate respiratory support step-down showed a success rate of 38% [24]. Comparing only infants on mechanical ventilation, the proportion of infants successfully extubated after dexamethasone treatment decreased from 52% with the first course (before 28 days) to 35% with the second course (after 28 days) [24]. The different rate of steroid success between these two studies could not be explained.

### 3.7. Treatment Timing

One epidemiological study investigating different treatment timing found higher adjusted odds ratios for death and severe BPD if steroids were initiated before 4 and after 8 weeks of life and suggested an optimal timing after 28 and before 50 days of life [26].

Three retrospective studies described treatment after 28 days of life and compared it to earlier treatment. The meta-analysis showed that treatment after 28 days of life was associated with a higher incidence of home oxygen (low quality of evidence) but lower mortality (very low quality of evidence). No effects could be found on the duration of oxygen dependency or length of stay (Table S4).

## 4. Discussion

This systematic review and meta-analysis sought to ascertain the validity of steroid treatment for established BPD, with the objectives of providing clinicians with a comprehensive evidence foundation and informing future research.

Our meta-analysis showed that despite having a good rate of success in terms of oxygen decrease, more clinically relevant outcomes, such as total duration of supplemental oxygen, length of stay, and mortality, were not affected by the use of steroids after 28 days of life. Interestingly, despite steroid treatment being regularly used, only one study reported the incidence of this treatment for established BPD [22]. Other authors from two independent level IV centers (one of them with specific expertise in BPD) described steroid administration as a standard treatment for severe BPD [30,31]. This approach could be explained by the fact that anecdotally and unpredictably, some BPD patients seem to respond well to steroids. Therefore, despite not being supported by robust evidence, steroids are often administered in neonatal practice as a last resort for BPD, in order not to miss those responders. Identifying potential steroid responders is paramount to targeting BPD therapeutic approaches. In addition to the inflammation that is present in the initial stage of the disease, the chronic phase of BPD is also characterized by structural changes, such as alveolar simplification, interstitial and peribronchial fibrosis, vascular changes, and airway abnormalities [9]. These alterations are less prone to being modified by steroids and may be present in different degrees in the various BPD phenotypes [9]. Therefore, it is plausible that when treating established BPD, patient selection for steroid therapy, based on disease phenotypes rather than disease severity, becomes paramount to boosting treatment success [9]. The attempt to identify steroid responders by clinical variables was not proven successful by the meta-analysis of the three studies investigating the factors associated with treatment response. In our analysis, the only factor that was significantly associated with steroid success after 28 days of life was treatment timing. However, this variable does not improve therapeutic precision in terms of patient selection. Artificial intelligence (AI)-assisted predictive models could potentially shine light on the multifactorial etiopathogenesis and phenotype characterization of BPD [39]. Lung ultrasound may be a valuable bedside asset in providing differential diagnoses for interstitial lung BPD phenotypes and predicting which patients may better respond to steroids [40].

Identifying patients who would respond to targeted treatments, including steroids, is a priority in the design of statistically and clinically valid studies.

The drug safety profile of steroids in the available literature supports the design of future RCTs in patients with established BPD. The only consistent steroid side effects noted were increased blood pressure and decreased weight gain during treatment, although neither persisted up to discharge. One study reported concern about brain volume after steroid treatment [28]. However, the study results may be biased, as steroid-treated infants were smaller and ventilated for a longer period. Mechanical ventilation is an independent risk factor for developmental delay in low-birth-weight preterm infants with every additional day of mechanical ventilation being strongly associated with an increased risk of poor neurodevelopmental outcomes and decreased brain volume [41].

A major limitation of this systematic review is the low number of eligible studies. However, the meta-analysis of RCTs explored well-designed double-blind studies, generating moderate quality of evidence for efficacy outcomes. One important obstacle to the meta-analysis was the fact that several potentially eligible studies enrolled patients treated with steroids before and after 28 days of life, including and analyzing together not only patients suffering from different severity degrees of BPD but also patients who had not developed any form of the condition. These patients were grouped by the definition of “chronically ventilated neonates” in most of the studies that were screened and excluded from our meta-analysis because treatment timing did not meet the inclusion criteria for all patients. However, there is no definition of chronically ventilated patients in neonatology, especially for preterm infants.

Another limitation of this meta-analysis is that due to insufficient studies meeting the inclusion criteria, it was impossible to run the pre-planned subgroup analysis. The included studies differed significantly in dosage, duration, and type of steroids, and these differences could not be accounted for.

A further limitation of this meta-analysis is the inclusion of different severity forms of BPD in the same analysis because of mixed data and the low number of studies. We also combined results from studies addressing inhaled and systemic steroids. However, we report subgroup analyses of separate data regarding the route of administration.

The variation in outcome measures used to define therapy success is a noted limitation of studies to date, further highlighting the importance of agreement on definitions in neonatology.

## 5. Conclusions

To the best of our knowledge, this is the first systematic review to examine available evidence on the use of steroids for the treatment of established BPD, from all sources including case series to randomized trials. The efficacy of steroids in reducing the supplemental oxygen need is consistent among the studies, independently of their design. However, the effectiveness of steroids in producing more compelling clinical outcomes could not be proved by the available studies.

The observed drug safety profile of steroids supports the design of future RCTs in patients with established BPD. Future studies should focus on patient selection to enroll those who manifest a possible late inflammatory phenotype of established BPD, investigate clinically important outcomes, and agree on a standardized definition for inclusion criteria.

## Figures and Tables

**Table 1:** Design of the included studies.

Study (First Author and Publication Year)	Country	Number of Sites	Study Design	Steroid Regimen			Inclusion Criteria	Number of Patients * (Study/Control)
				Drug/Control	Cumulative Dose	Route		
Noble-Jamieson, 1989 [18]	UK	Single	Double-blind RCT	Dexamethasone vs. placebo	5.95 mg/kg, over 21 days	Oral or IV	FiO_2_ > 0.30 after 4 weeks of age	18 (9/9)
Harkavy, 1989 [17]	USA	Single	Double-blind RCT	Dexamethasone vs. placebo	8 mg/kg, over at least 2 weeks	Oral or IV	Ventilator and oxygen dependency at 30 days of age	21 (9/12)
Beresford, 2002 [19]	UK	Single	Double-blind RCT	Fluticasone propionate vs. placebo	182.5 mg, over 1 year	Inhalation	Supplementary oxygen at 36 PMA	30 (15/15)
Dugas, 2005 [20]	Canada	Multi-center (2 centers)	Double-blind RCT	Fluticasone propionate vs. placebo	6.125 mg over 21 days; dose was doubled for infants >1200 g	Inhalation	28–60 days of life at treatment, FiO_2_ ≥ 0.25, pCO_2_ ≥ 45 mm Hg, chest radiograph compatible with BPD	32 (16/16)
Kugelman, 2017 [21]	Israel	Multi-center (5 centers)	Double-blind RCT	Beclomethasone vs. placebo	18 mg over 3 months	Inhalation	GA < 32 weeks, FiO_2_ ≥ 0.30 and/or positive pressure support with any FiO_2_ at 36 weeks PMA	38 (18/20)
Bhandari, 2008 [22]	USA	Single	Prospective cohort study	Prednisolone vs. no treatment	16 mg/kg over 14 days	Oral	Oxygen dependence at 36 weeks PMA	385 (131/254)
Bauer, 2009 [23]	Germany	Single	Prospective cohort study	Budesonide vs. no treatment	2.8 mg/kg, over 4 weeks	Inhalation	GA 25–31 weeks, supplemental oxygen in the first 28 days of life, signs of BPD	30 (10/20)
Cuna, 2018 [25]	USA	Single	Retrospective cohort study	Dexamethasone (late/early treatment)	0.72–0.89 mg/kg, over 7–10 days	IV	Preterm infants treated with postnatal steroids for BPD	55 (30/25)
Cuna, 2021 [24]	USA	Single	Retrospective cohort study	Dexamethasone (1 course vs. 2 courses)	0.72–0.89 mg/kg, over 7–10 days	IV	GA < 30 weeks received treatment with 1 or 2 courses of steroids for BPD	132 (52/80)
Harmon, 2020 [26]	USA	Single	Retrospective cohort study	Dexamethasone/hydrocortisone (late vs. early treatment)	Not specified	Not specified	GA < 27 weeks, received steroids between 8 days of life and <36 weeks PMA	951 (531/420)
Kwok, 2023 [27]	UK	Multi-center (185 centers)	Retrospective cohort study	Dexamethasone (late vs. early treatment)	400 mcg/die for 70.8 ± 44.2 days	Not specified	GA < 32 weeks and received 2 courses of steroids	1734 (636/10980)
Parikh, 2006 [28]	USA	Single	Retrospective case–control study	Dexamethasone vs. no treatment	2.8 mg/kg, over 7 days	Oral or IV	ELBW, receiving steroids after 4 weeks of life	41 (11/30)
Tiong, 2020 [29]	Taiwan	Single	Retrospective cohort study	Budesonide vs. no treatment	Not specified	Inhalation	ELBW preterm infants, ventilator dependence > 28 days with FiO_2_ > 0.60/PIP > 14 cmH_2_O	115 (64/51)
Brundage, 1992 [35]	Canada	Single	Pre–post study	Dexamethasone	3.5 mg/kg over 7 days	IV	BW < 1250 g with BPD, ventilator dependency at 3 weeks of age and failure to wean	7
Dassios, 2019 [32]	UK	Single	Retrospective cohort	Dexamethasone	2.7 mg/kg over 9 days	IV	GA < 30 weeks, ventilator dependency and FiO_2_ > 0.60	15
Linafelter, 2019 [30]	USA	Single	Retrospective cohort	Prednisolone or metylprednisolone	64.8 ± 41 mg/kg over 77 ± 38.3 days	Oral or IV	Infants dependent on CPAP or mechanical ventilation past 36 weeks PMA who received ≥30 days of therapy	43
Liviskie, 2021 [31]	USA	Single	Retrospective cohort	Prednisolone or metylprednisolone	Not specified	Oral or IV	Infants who received ≥30 days of therapy	40
Mizobuchi, 2001 [36]	Japan	Multi-center (2 centers)	Pre–post study	Dexamethasone	2.3 mg/kg over 7 days	Not specified	Infants dependent on ventilator for 28 days or longer	22
Tanney, 2011 [33]	UK	Single	Retrospective cohort	Dexamethasone	0.24 mg/kg over 9 days	Not specified	GA 23–26 weeks, deemed ventilator-dependent	9 *
Zhu H, 2021 [34]	China	Single	Prospective cohort	Dexamethasone	0.89 mg/kg over 10 days	IV	GA < 32, PMA > 36 w, and oxygen dependency > 28 days	30

Abbreviations: bronchopulmonary dysplasia (BPD); birth weight (BW); gestational age (GA); extremely low birth weight (ELBW); interquartile range (IQR); intravenously (IV); post-menstrual age (PMA). * Only patient treated after 28 days were included.

**Table 2:** Characteristics of the patients enrolled in the included studies.

Study (First Author and Publication Year)	GA (Weeks)		BW (Grams)		PMA at Treatment (Weeks)		Days at Treatment	
	Study Group	Control Group	Study Group	Control Group	Study Group	Control Group	Study Group	Control Group
Randomized controlled trials								
Noble Jamieson, 1989 [18]	28.0 ± 1.8	27.8 ± 2	1090 ± 199	1066 ± 196	33 ± 1.5 *	33 ± 1.7 *	36 ± 8	38 ± 10
Harkavy 1989 [17]	26.1 ± 2.0	25.9 ± 1.0	857 ± 183	772 ± 81	31 ± 2.6 *	30.7 ± 1.5 *	34.3 ± 2.8	34.1 ± 1.8
Beresford, 2002 [19]	26.8 ± 0.9	28.2 ± 0.9	885 ± 87	1123 ± 175	39.8 ± 1.4	39.8 ± 1.4	91 ± 9.9 *	81.2 ± 9 *
Dugas, 2005 [20]	27 ± 2.3	27.2 ± 1.7	995 ± 439	926 ± 251	33.4 ± 1.6 *	33.7 ± 1.4 *	44.8 ± 11	45.4 ± 10
Kugelman, 2017 [21]	26.4 ± 2.1	26.9 ± 1.9	838 ± 234	835 ± 263	36.2 ± 0.3	36.2 ± 0.4	68.6 ± 11 *	65.1 ± 10 *
Non-randomized cohort studies								
Bhandari, 2008 [22]	26.9 ± 2.2	27.2 ± 2.2	978 ± 342	1005 ± 357	38 ± 3.8	NA	77 ± 20 *	NA
Bauer, 2009 [23]	28.0 ± 1.9	27.0 ± 1.8	887 ± 210	841 ± 144	32.3 ± 7.2 *	NA	30 ± 1.6	NA
Parikh, 2006 [28]	25.1 ± 1.0	26.2 ± 1.6	740 ± 118	808 ± 146	NR	NR	NR	NR
Tiong, 2020 [29]	25.3 ± 1.2	25.3 ± 1.2	748 ± 133	771 ± 117	NR	NR	NR	NR
Single-arm studies								
Brundage, 1992 [35]	26.9 ± 0.7	NA	971 ± 86	NA	31.6 ± 0.6 *	NA	32.9 ± 4.8	NA
Dassios, 2019 [32]	25.7 ± 0.7	NA	795 ± 74.4	NA	34.5 ± 1.2	NA	61.6 ± 7.1	NA
Linafelter, 2019 [30]	26.1 ± 2.3	NA	729 ± 274	NA	43.3 ± 7.8	NA	123.2 ± 49	NA
Liviskie, 2021 [31]	26.5 ± 2.5	NA	846 ± 353	NA	41.7 ± 5	NA	107 ± 35	NA
Mizobuchi, 2001 [36]	25.8 ± 1.3	NA	750 ± 149	NA	32.1 ± 1.3	NA	45 ± 11	NA
Tanney, 2011 ** [33]	25.6 ± 1.4	NA	690 ± 127	NA	30 ± 1.2	NA	39 ± 9	NA
Zhu [34]	31.2 ± 1.4	NA	1340 ± 250	NA	NR	NA	NR	NA
Steroid treatment timing: late (after 28 days of life) vs. early (before 28)								
Study (First Author, Publication Year)	GA (Weeks)		BW (Grams)		PMA at Treatment (Weeks)		Days at Treatment	
	Early Steroid	Late Steroid	Early Steroid	Late Steroid	Early Steroid	Late Steroid	Early Steroid	Late Steroid
Cuna, 2018 [25]	24.9 ± 1.4	25.2 ± 1.2	729 ± 190	751 ± 135	28.2 ± 1.5	30.2 ± 1.3	23 ± 4	35 ± 4
Harmon, 2020 [26]	24.9 ± 1.0	24.9 ± 1.0	669 ± 132	687 ± 136	27.7 ± 1.3	31.4 ±2.0	21 ± 2	44 ± 3
Kwok, 2023 [27]	25.5 ± 0.7	24.3 ± 0.6	718.7 ± 38.7	676.8 ± 30.4	NR	NR	NR	NR
Steroid treatment: repeated dose								
Study (First Author, Publication Year)	GA (Weeks)		BW (Grams)		PMA at Treatment (Weeks)		Days at Treatment	
	One Course	Repeat Course	One Course	Repeat Course	One Course	Repeat Course	One Course	Repeat Course
Cuna 2021 [24]	25.1 ± 1.3	25.4 ± 2.0	737.6 ± 186.2	710.3 ± 198.0	30.5 ± 3	35.7 ± 4	36.4 ± 18	72.4 ± 25

Abbreviations: birth weight (BW); gestational age (GA); not applicable (NA); not reported (NR); post-menstrual age (PMA). * Derived data. ** Only patients treated after 28 days were included.

**Table 3:** Summary of findings of included trials with subgroup analysis according to administration route.

Outcomes	Studies		Statistics		Quality of Evidence	
	Number	RR/OR or MD (95% CI)	I^2^	P (RR, OR, MD)	ROB2/ROBIN	Certainty (GRADE)
Daily fall in FiO_2_ over 10 days ^#^ (MD in percentage per day)	2 RCTs [17,18]	1.6 (0.25 to 2.95)	0	<0.001	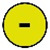 Some concerns	⊕⊕⊕⊖ Moderate
Oxygen dependency (MD in days)	5 RCTs [17,18,19,20,21]	6.4 (−12 to 25.1)	40	0.51	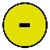 Some concerns	⊕⊕⊕⊖ Moderate
Systemic	2 RCTs [17,18]	−20.8 (−70 to 28.5)	0	0.41	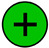 Low	⊕⊕⊕⊖ Moderate
Inhaled	3 RCTs [19,20,21]	11.3 (−9.3 to 32)	49	0.14	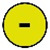 Some concerns	⊕⊕⊕⊖ Moderate
Length of stay (MD in days)	3 RCTs [17,20,21]	8.2 (−8.5 to 25)	0	0.8	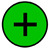 Low	⊕⊕⊕⊖ Moderate
Systemic	1 RCT [17]	−8 (−99.5 to 83.5)	NA	0.82	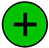 Low	Insufficient
Inhaled	2 RCTs [20,21]	8.75 (−8.3 to 25.8)	35	0.7	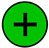 Low	⊕⊕⊕⊖ Moderate
Mortality, RR	4 RCTs [17,18,20,21]	0.9 (0.24 to 3.6)	0	0.3	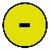 Some concerns	⊕⊕⊕⊖ Moderate
Systemic	2 RCT [17,18]	0.82 (0.15 to 4.5)	0	0.5	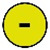 Some concerns	⊕⊕⊕⊖ Moderate
Inhaled	2 RCT [20,21]	1.16 (0.12 to 10.6)	0	0.4	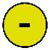 Some concerns	⊕⊕⊕⊖ Moderate
Weight gain during treatment (MD in grams/day) ^#^	2 RCTs [17,18]	−9.2 (−11.7 to −5.6)	0	<0.001	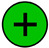 Low	⊕⊕⊕⊖ Moderate
Overall weight gain (MD in grams/day)	3 RCTs [17,20,21]	2.4 (−0.3 to 6.3)	0	0.078	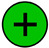 Low	⊕⊕⊕⊖ Moderate
Systemic	1 RCTs [17]	3 (−0.4 to 6.4)	0	0.081	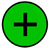 Low	Insufficient
Inhaled	2 RCTs [20,21]	1.4 (−3.4 to 6.3)	0	0.56	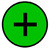 Low	⊕⊕⊕⊖ Moderate
Systolic blood pressure (MD in mmHg) *	2 RCTs [20,21]	6.8 (4.63 to 8.9)	0	<0.001	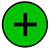 Low	⊕⊕⊕⊖ Moderate
Adrenal insufficiency (MD in cortisol levels) *	2 RCTs [20,21]	2.3 (−43 to 47.6)	88	0.92	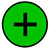 Low	⊕⊖⊖⊖ Very low
Hyperglycemia, RR	3 RCTs [17,18,20]	2.8 (0.5 to 18)	60	0.26	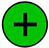 Low	⊕⊖⊖⊖ Very low
Systemic	2 RCT [17,18]	4 (0.48 to 37)	48	0.2	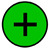 Low	⊕⊖⊖⊖ Very low
Inhaled	1 RCT [20]	1.1 (0.04 to 35)	NA	0.92	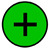 Low	Insufficient
Infections, RR ^#^	2 RCTs [17,18]	0.7 (0.26 to 1.92)	0	0.35	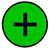 Low	⊕⊕⊕⊖ Moderate
Total brain volume ^#^ (MD in percentage)	1 case control [28]	−10 (−0.1 to −19)	NA	0.03	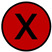 High	Insufficient
Cerebral palsy, OR *~	1 cohort study [29]	156 (0.89–27399)	NA	0.6	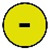 Some concerns	Insufficient
NDI, OR *~	1 cohort study [29]	1.70 (0.55 to 5.27)	NA	0.36	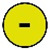 Some concerns	Insufficient

^#^ Systemic steroids only. * Inhaled steroids only. ~ Adjusted OR. Abbreviations: risk ratio (RR); mean difference (MD); odds ratio (OR); randomized control trial (RCT); not applicable (NA); neurodevelopmental impairment (NDI). Statistics section of the table is highlighted in light blue. Grading of evidence and risk of bias is color-coded.

**Table 4:** Percentage and criteria for success reported in the included studies.

Reference	Criteria for Steroid Success	Percentage	Success/Total Number
Bhandari, 2008 [22]	Wean off oxygen	62	82/131
Cuna, 2021 [24]	Successful extubation	37	12/34
Dassios, 2019 [32]	Successful extubation	93	14/15
Harkavy, 1989 [17]	Successful extubation	100	9/9
Kwok, 2023 [27]	Successful extubation within 14 days of starting steroids	78.6	591/843
Linafelter, 2019 [30]	Wean off oxygen	63	82/131
Mizobuchi, 2001 [36]	Successful extubation	100	22/22
Tanney, 2011 [33]	Successful extubation	88.8	8/9
Zhu, 2021 [34]	Wean off oxygen/self-ventilating in room air	33	10/30

## Data Availability

All data generated or analyzed during this study are included in this article [and its Supplementary Material Files]. Further enquiries can be directed to the corresponding author.

## Supplementary Materials

The following supporting information can be downloaded at: https://www.mdpi.com/article/10.3390/children12091238/s1, Figure S1. Risk of bias of the studies for which meta-analysis was performed. Figure S2. Sensitivity analysis with one study removed at a time. Table S1. GRADE evidence profile table for included randomized controlled trials. Table S2. GRADE evidence profile table for studies comparing steroid treatment before and after 28 days of life. Table S3. Meta-analysis of factors associated with success of steroid therapy. Table S4. Summary of findings for different timing of steroid administration (before and after 28 days).
