# Supplementary material for: Steroid Use for Established Bronchopulmonary Dysplasia: A Systematic Review and Meta-Analysis
Source: Children (Basel). 2025 Sep 16;12(9):1238. doi: 10.3390/children12091238 (PMC12468976; doi:10.3390/children12091238)
Supplement: Supplementary file 1 [file children-12-01238-s001.zip › children-3833367-supplementary.pdf]

STEROID USE FOR ESTABLISHED BRONCHOPULMONARY DYSPLASIA: A SYSTEMATIC REVIEW AND META-ANALYSIS

SUPPLEMENTAL MATERIAL

AMANDEMENTS

When this meta-analysis was designed, we aimed to include only studies that treated infants with moderate and severe BPD from 36 weeks PMA [1], in agreement with recent definitions of BPD [2,3]. However, most of the studies on steroids for established BPD include a mixed set of patients with mild, moderate and severe BPD, according to the 2001 National Institute of Child Health and Human Development (NICHD) workshop. The NICHD 2001 workshop define BPD as oxygen dependency for at least 28 days of life, stratifying the cases in 3 levels of severity: mild (if the infant does not need oxygen at 36 weeks PMA) moderate and severe (depending on how much oxygen the infant needs at 36 weeks PMA) [1]. We could not obtain separate data for infants treated with steroids exclusively after 36 weeks PMA from the authors. We decided to include patient treated with steroids for established mild BPD, in addition to the patients suffering from the moderate and severe forms of the disease.

Search strategy

The search query, developed by a librarian (KW), was as follows:

(steroid[tiab] OR steroids[tiab] OR betamethasone[tiab] OR dexamethasone[tiab] OR hydrocortisone[tiab] OR prednisolone[tiab] OR glucocorticoids[tiab] OR budesonide[tiab] OR methylprednisolone[tiab] OR “Steroids"[Mesh:NoExp] OR “Betamethasone"[Mesh] OR “Dexamethasone"[Mesh] OR "Hydrocortisone"[Mesh] OR “Prednisolone"[Mesh] OR ”Glucocorticoids”[Mesh] OR "Budesonide"[Mesh:NoExp] OR "Methylprednisolone"[Mesh]) AND (infant[tiab] OR infants[tiab] OR newborn[tiab] OR newborns[tiab] OR neonate[tiab] OR

neonates[tiab] OR neonatal[tiab] OR postnatal[tiab] OR "Infant"[Mesh] OR "Intensive Care Units, Neonatal"[Mesh]) AND ("bronchopulmonary dysplasia"[tiab] OR BDP[tiab] OR "respiratory distress syndrome"[tiab] OR "ventilator-induced lung injury"[tiab] OR "ventilator-induced lung injuries"[tiab] OR "chronic lung disease"[tiab] OR "chronic lung diseases"[tiab] OR CLD[tiab] OR "chronic lung injury"[tiab] OR "chronic lung injuries"[tiab] OR "artificial respiration"[tiab] OR "artificial respirations"[tiab] OR "mechanical respiratory support"[tiab] OR "mechanical ventilation"[tiab] OR "mechanical ventilations"[tiab] OR ”non-invasive ventilation”[tiab] OR ”non-invasive ventilations”[tiab] OR ”noninvasive ventilation”[tiab] OR ”noninvasive ventilations”[tiab] OR "nasal intermittent ventilation"[tiab] OR NIV[tiab] OR "nasal intermittent positive pressure ventilation"[tiab] OR NIPPV[tiab] OR "continuous positive airway pressure"[tiab] OR CPAP[tiab] OR "high flow nasal cannula"[tiab] OR "oxygen inhalation therapy"[tiab] OR "oxygen inhalation therapies"[tiab] OR "Infant, Premature, Diseases"[Mesh:NoExp] OR "Bronchopulmonary Dysplasia"[Mesh] OR "Respiratory Distress Syndrome, Newborn"[Mesh] OR "Neonatal Respiratory Distress Syndrome"[Mesh] OR "Ventilator-Induced Lung Injury"[Mesh:NoExp] OR "Respiration, Artificial"[Mesh:NoExp] OR "Noninvasive Ventilation"[Mesh] OR "Continuous Positive Airway Pressure"[Mesh] OR "Oxygen Inhalation Therapy"[Mesh:NoExp]) NOT (review[pt] OR "systematic review"[pt] OR "preclinical study"[ti]) NOT ("animals"[mesh] NOT "humans"[mesh]).

REFERENCES

1. *Bronchopulmonary dysplasia.* Jobe AH, Bancalari E. 7, 2001, Am J Respir Crit Care Med., Vol. 163, pp. 1723-1729.

2. *Bronchopulmonary Dysplasia: Executive Summary of a Workshop.* Higgins RD, Jobe AH, Koso-Thomas M, et al. 2018, J Pediatr. , Vol. 197, pp. 300-308.

3. *The Diagnosis of Bronchopulmonary Dysplasia in Very Preterm Infants. An Evidence-based Approach.* Jensen EA, Dysart K, Gantz MG, et al. 6, 2019, Am J Respir Crit Care Med, Vol. 200, pp. 751-759.

FIGURES AND TABLES

A

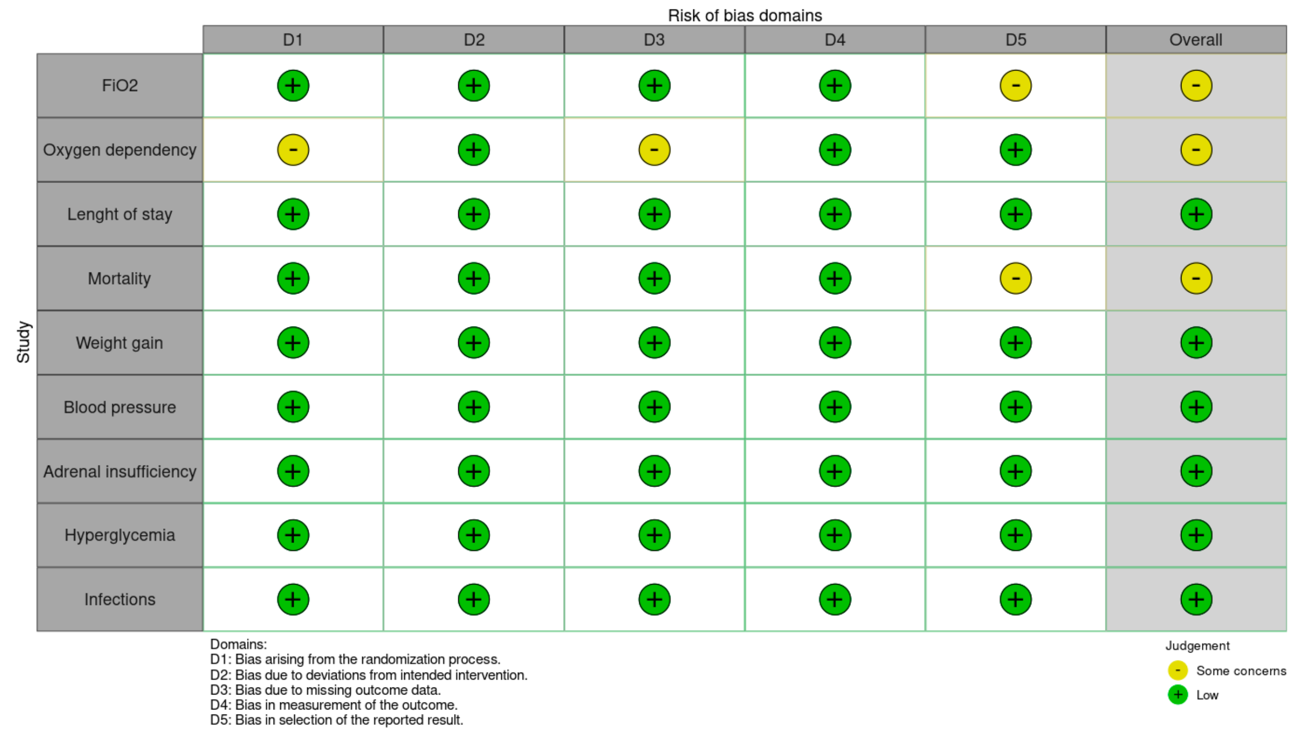

B

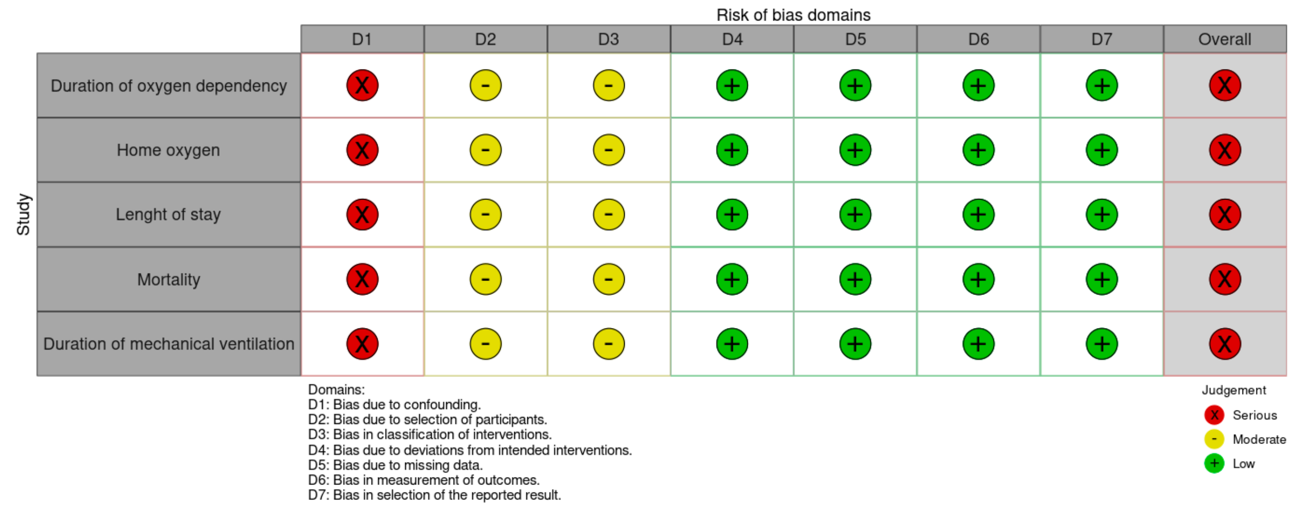

Figure S1. Risk of bias of the studies for which meta-analysis was performed. Randomized trials (a) and non-randomized trials investigating steroids before and after 28 days of life (b)

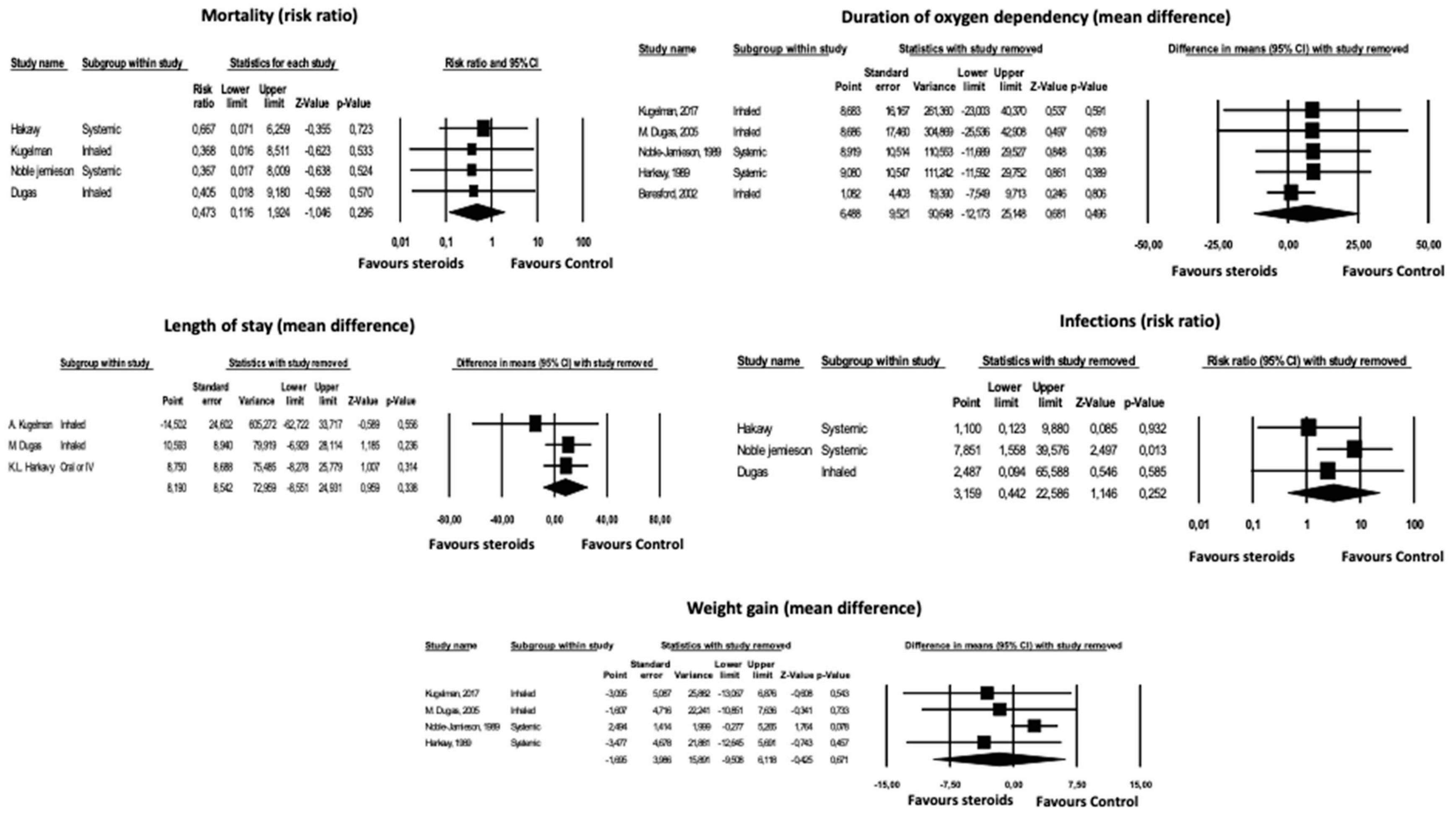

Mortality (risk ratio) — Statistics for each study

Study name	Subgroup within study	Risk ratio	Lower limit	Upper limit	Z-Value	p-Value
Hakawy	Systemic	0,667	0,071	6,259	-0,355	0,723
Kugelman	Inhaled	0,368	0,016	8,511	-0,623	0,533
Noble jemieson	Systemic	0,367	0,017	8,009	-0,638	0,524
Dugas	Inhaled	0,405	0,018	9,180	-0,568	0,570
		0,473	0,116	1,924	-1,046	0,296

Duration of oxygen dependency (mean difference) — Statistics with study removed

Study name	Subgroup within study	Point	Standard error	Variance	Lower limit	Upper limit	Z-Value	p-Value
Kugelman, 2017	Inhaled	8,683	16,167	261,360	-23,003	40,370	0,537	0,591
M. Dugas, 2005	Inhaled	8,686	17,460	304,869	-25,536	42,908	0,497	0,619
Noble-Jamieson, 1989	Systemic	8,919	10,514	110,553	-11,689	29,527	0,848	0,396
Harkavy, 1989	Systemic	9,080	10,547	111,242	-11,592	29,752	0,861	0,389
Beresford, 2002	Inhaled	1,082	4,403	19,390	-7,549	9,713	0,246	0,806
		6,488	9,521	90,648	-12,173	25,148	0,681	0,496

Length of stay (mean difference) — Statistics with study removed

	Subgroup within study	Point	Standard error	Variance	Lower limit	Upper limit	Z-Value	p-Value
A. Kugelman	Inhaled	-14,522	24,602	605,272	-62,722	33,717	-0,589	0,556
M Dugas	Inhaled	10,593	8,940	79,919	-6,929	28,114	1,185	0,236
K.L. Harkavy	Oral or IV	8,750	8,688	75,485	-8,278	25,779	1,007	0,314
		8,190	8,542	72,959	-8,551	24,931	0,959	0,338

Infections (risk ratio) — Statistics with study removed

Study name	Subgroup within study	Point	Lower limit	Upper limit	Z-Value	p-Value
Hakawy	Systemic	1,100	0,123	9,880	0,085	0,932
Noble jemieson	Systemic	7,851	1,558	39,576	2,497	0,013
Dugas	Inhaled	2,487	0,094	65,588	0,546	0,585
		3,159	0,442	22,586	1,146	0,252

Weight gain (mean difference) — Statistics with study removed

Study name	Subgroup within study	Point	Standard error	Variance	Lower limit	Upper limit	Z-Value	p-Value
Kugelman, 2017	Inhaled	-3,095	5,087	25,882	-13,067	6,876	-0,608	0,543
M. Dugas, 2005	Inhaled	-1,607	4,716	22,241	-10,851	7,636	-0,341	0,733
Noble-Jamieson, 1989	Systemic	2,494	1,414	1,999	-0,277	5,265	1,764	0,078
Harkay, 1989	Systemic	-3,477	4,678	21,881	-12,645	5,691	-0,743	0,457
		-1,695	3,986	15,891	-9,508	6,118	-0,425	0,671

Figure S2. Sensitivity analysis with one study removed at a time.

Sensitivity analysis run outcomes of randomized trials that had at least three studies in the analysis.

QUALITY ASSESSMENT							GRADE SUMMARY OF FIDINGS	
No of studies	Design	Risk of bias	Inconsistency	Indirectness	Imprecision[3]	Others[4]	Certainty (overall score)	Importance
**EFFICACY OUTCOMES**								
**Outcome:** Fraction of inspired oxygen (FiO2)								
2	RCT	Some concern[1]	None	None	Serious (-1)	None	⊕⊕⊕⊖ Moderate	Important
**Outcome:** Duration of oxygen dependency								
5	RCT	Some concern[1]	Low[2]	None	Serious (-1)	None	⊕⊕⊕⊖ Moderate	Important
**Outcome:** Length of stay								
3	RCT	Low	None	None	Serious (-1)	None	⊕⊕⊕⊖ Moderate	Important
**Outcome:** Mortality								
4	RCT	Some concern[1]	None	None	Serious (-1)	None	⊕⊕⊕⊖ Moderate	Of most importance
**SAFETY OUTCOMES**								
**Outcome:** Overall weight gain								
3	RCT	Low	None	None	Serious (-1)	None	⊕⊕⊕⊖ Moderate	Of most importance
**Outcome:** Weight gain during treatment								
2	RCT	Low	None	None	Serious (-1)	None	⊕⊕⊕⊖ Moderate	Important

**Outcome:** Blood pressure								
2	RCT	Low	None	None	Serious (-1)	None	⊕⊕⊕⊖ Moderate	Important
**Outcome:** Adrenal insufficiency								
2	RCT	Low	Very serious[6] (-2)	Low	Serious (-1)	None	⊕⊖⊖⊖ Very low	Critical
**Outcome:** Hyperglycemia								
3	RCT	Low	Serious[6] (-1)	Low	Very Serious (-2)	None	⊕⊖⊖⊖ Very low	Important
**Outcome:** Infections								
2	RCT	Low	None	Low	Serious (-1)	None	⊕⊕⊕⊖ Moderate	Critical

Table S1. GRADE evidence profile table for included randomized controlled trials
Abbreviation: Randomized controlled trials (RCTs)
Footnotes.
[1] Overall ROB shows some concerns, but was not considered enough to downgrade the evidence
[2] Values above 0 but not considered significant enough to downgrade the evidence
[3] Optimal information size (OIS) was calculated for each outcomes and evidence was rated down if actual sample size for the specific outcomes was lower than the OIS
[4] Publication bias was not calculated because of low number of studies
[5] Possibly caused by route of administration

QUALITY ASSESSMENT						SUMMARY OF FIDINGS		
**No of studies**	**Design**	**Risk of bias**	**Inconsistency**	**Indirectness**	**Imprecision** [3]	**Others**[4]	**Certainty (overall score)**	**Importance**
**Outcome:** Duration of mechanical ventilation								
2	Non-RCT	Serious (-1)	Low [1]	None	None	None	⊕⊖⊖⊖ Very low	Critical
**Outcome:** Duration of oxygen dependency								
2	Non-RCT	Serious (-1)	Serious [2] (-1)	None	None	None	⊕⊖⊖⊖ Very low	Critical
**Outcome:** Home oxygen								
3	Non-RCT	Some concern	None	None	None	None	⊕⊕⊖⊖ Low	Critical
**Outcome:** Length of stay								
2	Non-RCT	Serious (-1)	Serious [2] (-1)	None	None	None	⊕⊖⊖⊖ Very low	Important
**Outcome:** Mortality								
3	Non-RCT	Serious (-1)	None	None	None	None	⊕⊖⊖⊖ Very low	Of most importance

Table S2. GRADE evidence profile table for studies comparing steroid treatment before and after 28 days of life
Abbreviation: non-Randomized controlled trials (non-RCTs)
Footnotes.
[1] Some inconsistency, not enough to grade down evidence
[2] No explanation for inconsistency found
[3] Optimal information size (OIS) was calculated for each outcomes and evidence was rated down if actual sample size for the specific outcomes was lower than the OIS
[4] Publication bias was not calculated because of low number of studies. No factors that could upgrade the quality of evidence was found

Factor	Success n (%)	Failure n (%)	Mean±SD or n (%) Success	Mean±SD Or n (%) Failure	MD or OR [95%CI]	I^2	Tau	P Value
GA [21,33]	92 (57.1)	69 (42.9)	29±1.65	29.25±2	-0.041 [-0.36;0.28]	0	0	0.84
Male gender [21,33]	92 (57)	69 (43)	56 (61)	41 (59)	1.09 [0.57;2.11]	0	0	0.77
Days at treatment [21,23]	94 (56.9)	71 (43.1)	70±19.2	75.5±25	-0.38 [-0.7;-0.06]	0	0	0.019
PMA at treatment [21,23]	94 (56.9)	71 (43.1)	36.3±2.8	37.3±3.3	-0.11 [-0.47;0.27]	12.8	0.11	0.53
pCO2 [21,23]	94 (56.9)	71 (43.1)	53.5±6	58.55±8.4	-0.4 [-1.96;0.52]	89	0.85	0.256

Table S3. Meta- analysis of factors associated with success of steroid therapy
Abbreviations: Confidence interval (CI); Gesational age (GA); Mean difference (MD); Odds ratio (OR); partial Carbon diaxide pressure (pCO2); post-menstrual age (PMA); standard deviation (SD)

Outcome	Studies, patients RR	Absolute effect estimate		Certainty of the evidence (Quality of evidence)	Plain language summary
		Early Steroids	Late steroids		
**MV duration (days)**	2 retrospective cohort studies [24,25] *1006 patients*	**46.7** Days (mean)	**52.9** Days (mean)	⊕⊖⊖⊖ **Very low** Seriuos ROB (-1)	It is **uncertain** whether late as compared early steroids decrease the *duration of MV* — Uncertain
		Difference **6.2 days fewer** (95% CI from 9.2 to 3.2 fewer)			
**Oxygen dependency (days)**	2 retrospective cohort studies [24,25] *1006 patients*	**106** Days (mean)	**123** Days (mean)	⊕⊖⊖⊖ **Very low** Seriuos ROB (-1) Seriuos imprecision (-1)	It is **uncertain** whether late as compared early steroids decrease the *duration of oxygen dependency* — Uncertain
		Difference **17 days fewer** (95% CI 46 fewer to 13 more)			
**Home oxygen**	2 retrospective cohort studies [24,25] *1006 patients* **OR 0.56 (0.44 to 0.72)**	**42** per 100	**56** per 100	⊕⊕⊖⊖ **Low**	**Probably** late as compared early steroids **slightly increase** the *discharge on oxygen* — Favors early steroids
		Difference **14 fewer per 100** (95% CI 20 to 8 fewer)			
**Mortality**	3 retrospective cohort studies [24–26] *3104 patients* **OR 2.56 (2.04 to 3.2)**	**20** per 100	**10** per 100	⊕⊖⊖⊖ **Very low** Seriuos ROB (-1)	It is **uncertain** whether late as compared early steroids decrease *mortality* — Uncertain
		Difference **10 more per 100** (95% CI 8 to 13 more)			
**Length of stay (days)**	2 retrospective cohort studies [24,25] *1006 patients*	**131.2** Days (mean)	**134.9** Days (mean)	⊕⊖⊖⊖ **Very low** Seriuos ROB (-1)	It is **uncertain** whether late as compared early steroids decrease *length of stay* — Uncertain
		Difference **3.7 days less** (95% CI 9.8 fewer to 2.3 more)			

Table S4. Summary of findings for different timing of steroid administration (before and after 28 days)
Cohort and case-control studies start from a low confidence (low quality of the evidence). When the certainty of the evidence is very low, no conclusion can be made and the evidence is uncertain.Abbreviations: Confidence interval (CI); Mean difference (MD); Mechanical ventilation (MV); Odds ratio (OR); Risk of BIAS
